# Artemisinin combination therapy fails even in the absence of *Plasmodium falciparum kelch13* gene polymorphism in Central India

**DOI:** 10.1038/s41598-021-89295-0

**Published:** 2021-05-11

**Authors:** Sabyasachi Das, Amrita Kar, Subhankar Manna, Samaresh Mandal, Sayantani Mandal, Subhasis Das, Bhaskar Saha, Amiya Kumar Hati

**Affiliations:** 1 Department of Physiology, Faculty of Medicine, Manipal University College Malaysia, Jalan Batu Hampar, Bukit Baru, 75150 Melaka, Malaysia; 2 Department of Physiology, Faculty of Medicine, Lincoln University College, Petaling Jaya, Selangor, Malaysia; 3 Department of Human Physiology, Vidyasagar University, Midnapore, West Bengal India; 4 Department of Biotechnology, Centre for Research in Infectious Diseases, School of Chemical and Biotechnology, Sastra University, Thanjavur, Tamil Nadu India; 5 Medicare, Bhilai, Chhattisgarh India; 6 Lab 5, National Centre for Cell Science, Ganeshkhind, Pune, India; 7 Department of Medical Entomology and Parasitology, Calcutta School of Tropical Medicine, Kolkata, West Bengal India

**Keywords:** Antimicrobial resistance, Antiparasitic agents, Haplotypes, Genetic markers, Malaria

## Abstract

Artemisinin is the frontline fast-acting anti-malarial against *P. falciparum*. Emergence and spread of resistant parasite in eastern-India poses a threat to national malaria control programs. Therefore, the objective of our study is to evaluate the artesunate-sulfadoxine-pyrimethamine efficacy in Central India. 180 monoclonal *P. falciparum*-infected patients received standard ASSP therapy during August 2015–January 2017, soon after diagnosis and monitored over next 42-days. Artemisinin-resistance was assessed through in-vivo parasite clearance half-life (PC_1/2_), ex-vivo ring-stage survivability (RSA), and genome analysis of *kelch13* and other candidate gene (*pfcrt, pfmdr1, pfatpase 6, pfdhfr* and *pfdhps*). Of 180 *P. falciparum* positive patients, 9.5% showed increased PC_1/2_ (> 5.5 h), among them eleven isolates (6.1%) showed reduced sensitivity to RSA. In 4.4% of cases, parasites were not cleared by 72 h and showed prolonged PC_1/2_(5.6 h) (P < 0.005) along with significantly higher RSA (2.2%) than cured patients (0.4%). None of day-3 positive isolates contained the *pfkelch13* mutation implicated in artemisinin resistance. Parasite recrudescence was observed in 5.6% patients, which was associated with triple *dhfr*–*dhps* (A_16_I_51_R_59_N_108_I_164_–S_436_G_437_K_540_G_581_T_613_) combination mutation. Emergence of reduced sensitivity to artesunate-sulfadoxine-pyrimethamine, in central India highlighted the risk toward spread of resistant parasite across different parts of India. Day-3 positive parasite, featuring the phenotype of artemisinin-resistance without *pfkelch13* mutation, suggested *kelch13*-independent artemisinin-resistance.

## Introduction

Drug resistant *P. falciparum* is one of the major factors for death in malaria. 445,000 deaths and an estimated 216 million confirmed malaria cases—including an increase of about 5 million cases over and above what was recorded in 2015-were reported in 2016^1^. Malaria transmission in India potentially occurs through either *P. falciparum* or *P. vivax* infection^2,3^*.* In India, 844,558 malaria cases were reported in 2017, of which 529,530 cases were *P. falciparum* positive^4^. North-eastern states and central Indian states contributed 80% of the total cases^5^. Chhattisgarh, one of the states in central India, contributed the second highest malaria incidence in India over the years^6^. National vector borne disease control program (NVBDCP) had launched artemisinin-based combination therapy (ACT) to wipe-out the burden of chloroquine (CQ) and sulfadoxine-pyrimethamine (SP) resistant malaria in 2009^7–9^. Success of ACT depends on a combination of fast-acting short half-life artemisinin derivatives with late-acting longer half-life 4-aminoquinolines or antifolates^10^. Global mortality and morbidity associated with malaria were considerably reduced after the introduction of ACT, but emergence and subsequent spread of artemisinin-resistant parasites in the Greater Mekong sub-region had seriously threatened the global malaria control and elimination progress^11–14^. Artemisinin resistance is characterized by drug failure reflected in slow parasite clearance as assessed by increased in vivo parasite clearance half-life (PC_1/2_) along with reduced sensitivity of ex-vivo ring-stage parasites to artemisinin^15–17^. Genome based transfection studies proved the association of artemisinin resistance with *kelch*13 gene-polymorphism which was directly linked with increased ex-vivo ring-stage survivability and prolonged in-vivo PC_1/2_ (> 5.5 h)^18–21^. Previous genome-based reports suggested that polymorphism in *pfatpase6*, *pfmdr1, pfcrt* and *P. falciparum* ferredoxin (*pffd*) genes also exhibited potential role on artemisinin resistance^22–24^. In addition to this, presence of day-3 parasite above 10% of day-0 parasitemia indicated a potential treatment failure. The level of PCR adjusted cure rates after 28 days treatment follow-up below 90% against the WHO recommended first line therapy for uncomplicated *P. falciparum* called for its reassessment^25^.

Artesunate-sulfadoxine-pyrimethamine (ASSP) is the drug of choice against *P. falciparum* in India excepting the north-eastern states^9^. Declining efficacy of ASSP was previously reported from these states and eastern India^26–28^. The emergence and spread of partial artemisinin resistant parasites were previously reported from West Bengal-an eastern state of India^27,28^. Partial artemisinin resistance was associated with the failure of partner drugs in combination^14,24^. Prevalence of mutations in molecular markers (*pfdhfr* and *pfdhps*) associated with partner drug resistance (sulfadoxine–pyrimethamine) was previously reported from different parts of India including central India^8,29–33^. Therefore, the aim of this study was to critically examine the ASSP efficacy through in-vivo, ex-vivo and genome-wide variation studies in central India.

## Results

### Study population

**Figure 1:**
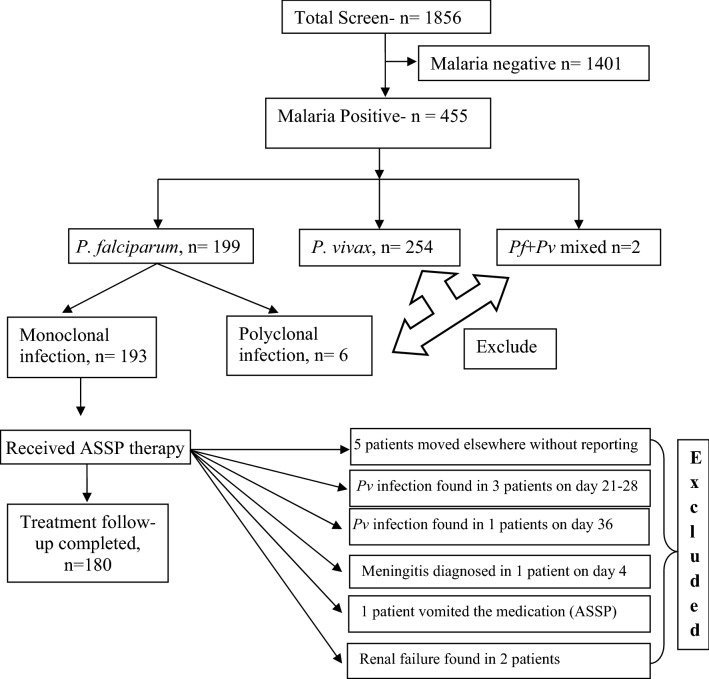
Schematic presentation of patient selection and entry criteria for randomization with ASSP. Monoclonal *P. falciparum* infections contained a single allelic form of infection i.e. either of mspI or mspII or *glurp.* Polyclonal infections along with *Pf, Pv* mixed infections were excluded. Only *P. falciparum* monoclonal infections were selected for the study. Patients with additional *P. vivax* co-infection during the follow-up scheduled were excluded. Patients, who had not completed the 42 days follow-up schedule, were eliminated.

A total of 1856 febrile patients were screened; of them, 199 patients (10.7%) were detected *P. falciparum* positive. Mean age of *P. falciparum*-infected persons was 30.7 years (range 8–69 years). 193 patients (193/199) were identified as monoclonal *P. falciparum* infection and received standard ASSP therapy. Patient characteristics on enrolment of the study were presented in Table 1. 180 patients (180/193) had successfully completed the 42 day’s follow-up. As we have only considered monoclonal *P. falciparum* infection, therefore patients with *P. vivax* infection (254/1856) and polyclonal *falciparum* (6/199) infections were excluded. A detailed information regarding patient selection (inclusion and exclusion criteria) were presented in Fig. 1.

**Table 1:** Patient characteristics on enrolment of the study.

Patient characteristics	Bhilai including durg
Age (year)	30.74 (95% CI 17–58)
Sex ratio (women/men)	73/107
Axillary temperature on day 0 (°C)	39.22 °C (95% CI 38.16–40.19)
Parasite density (parasite/µL)	44,152 (95% CI 9632–78,810)
Mean hemoglobin (g/dL)	12.3 (95% CI 10.2–14.8)
Hematocrit	Male: 47.1% (95% CI 45.8–49.5) Female: 38.8% (95% CI 37.4–41.7)

### Parasite clearance phenotype

**Figure 2:**
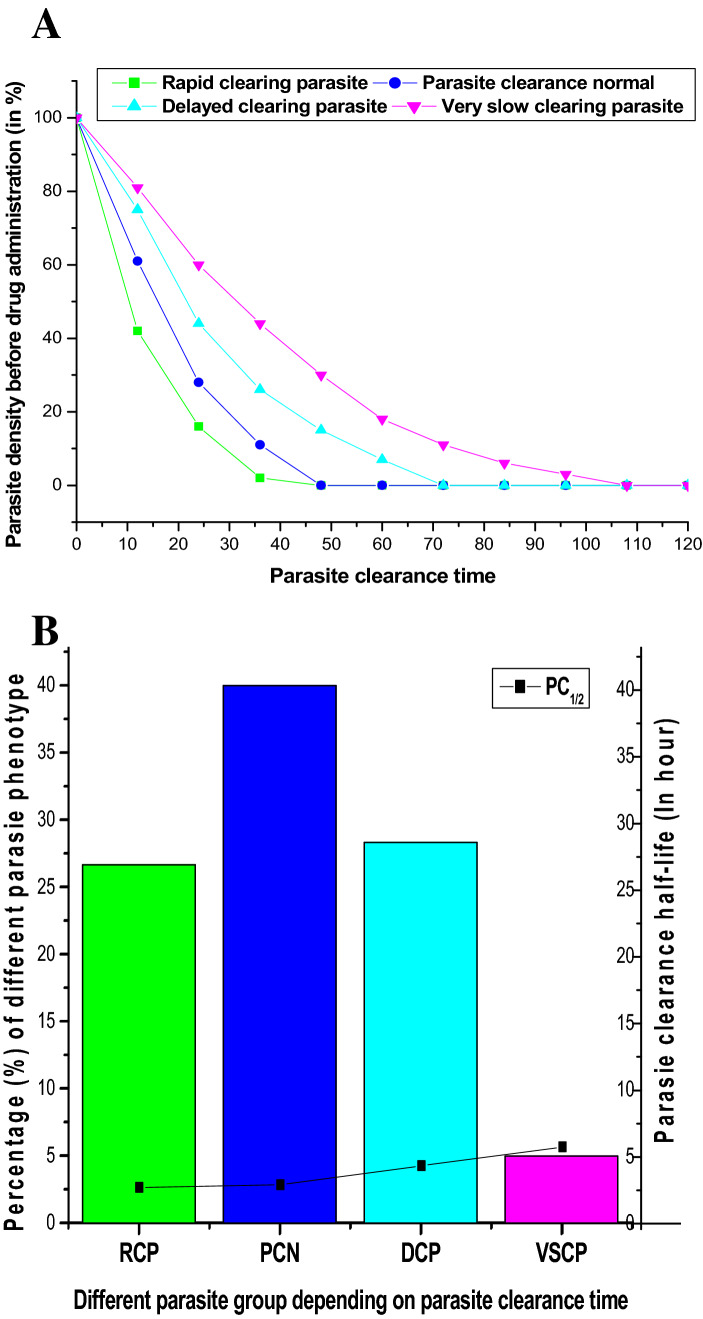
(**A**) Proportion of Parasite clearance phenotypes: we had classified four different parasite clearance phenotypes depending on the parasite clearance time. Parasites, those who cleared within 36 h of drug administration were classified as Rapid clearing parasite (RCP) whereas parasites cleared by 48 h of drug exposure were designated as Parasite clearance normal (PCN). In patients, those whose parasites were cleared by > 48 h to ≥ 72, were designated as Delayed clearing parasite (DCP). Parasites were not cleared after 72 h of drug exposure were designated as Very slow clearing parasite (VSCP). (**B**) Frequency of different Parasite clearance phenotypes in relation to PC_1/2_: Parasite clearance normal (PCN) phenotype (40%), was most prevalent followed by delayed clearing parasite (DCP; 28.88%) and rapid clearing parasite (RCP; 26.11%). Interestingly, 5% of isolates represented VSCP phenotypes. We found low median PC_1/2_ in RCP (2.6 h) and PCN phenotype (2.8 h). Higher PC_1/2_ was observed in DCP phenotype (4.2 h) while very high median PC_1/2_ was recorded in VSCP phenotype (5.6 h) which proved these phenotypes perhaps less sensitive to ASSP therapy.

Based on parasite clearance time (PCT), we classified four different parasite clearance phenotypes. We defined, PCT ≤ 36 h, as rapid-clearing parasite (RCP); PCT > 36– ≤ 48 h as parasite-clearance normal (PCN); PCT > 48– ≤ 72 h as delayed-clearing parasite (DCP); and PCT > 72 h as very slow-clearing parasite (VSCP) (Fig. 2A). We found low median PC_1/2_ in RCP (2.6 h; 95% Cl 2.3–3 h) and PCN phenotype (2.8 h; 95% Cl 2.4–3.2 h). We recorded very high median PC_1/2_ in VSCP phenotype (5.6 h; 95% Cl 5.5–5.7 h) and higher PC_1/2_ in DCP phenotype (4.2 h; 95% Cl 3.7–4.8 h) (Fig. 2B). 17 patients (9.5%) showed prolonged PC_1/2_ (> 5.5 h) (8/52, DCP; and 9/9 VSCP phenotype) (Table 2). We observed a significant statistical difference in PC_1/2_ among these four parasite phenotypes (Kruskal–Wallis Test, p = 0.0042).

**Table 2:** Distribution of different candidate genotype in relation to ASSP combination treatment.

No of isolates	*kelch* 13	*atpase6 *(263 + 431 + 623 + 6 30 + 769)	*pfmdr1* (86 + 184 + 1034 + 1042 + 1246)	*pfdhfr* (16 + 51 + 59 + 108 + 164)	*pfdhps* (436 + 437 + 540 + 581 + 613)	*pfcrt* (72–76 + 326 + 356)	PC_1/2_		Parasite clearance time				Recrudescence	Ex-vivo AS (RSA) sensitivity	
							< 5 h	> 5 h	≤ 36 h	> 36–≤ 48 h	> 48–≤ 72 h	> 72hETF	(LTF)	S	RS
21	Wild	LEAAS	NYSND	ANCSI	SAKAA	CVMNKNI	21	–	10	8	3	–	–	18	–
11	Wild	LEAAS	YYSND	ANCNI	SAKAA	CVMNTNI	11	–	7	2	2	–	–	10	–
6	Wild	LEAAS	NFSND	AICNI	SAKGA	SVMNTSI	6	–	2	4	–	–	–	4	–
16	Wild	LEAAS	NFSND	AICNI	SGKAA	SVMNTSI	15	1	5	6	5	–	–	14	–
7	Wild	LEAAS	YYSND	ANRNI	AAKAA	SVMNTNT	7	–	4	3	–	–	–	6	
24	Wild	LEAAS	YYSND	AICNI	AGKAA	SVMNTNT	23	1	8	11	4	1	2	20	1
3	Wild	LEAAS	NFSND	AICTI	SGKAA	SVMNTSI	3	–	1	2	–	–	–	3	–
17	Wild	LEAAS	YYSND	AICNI	SGKAA	CVIETNT	17	–	4	11	2	–	–	16	–
6	N29L	LEAAS	NFSND	AICNI	AGKAA	SVMNTST	6	–	4	1	1	–	1	6	–
10	Wild	LEAAS	YYSND	ANRNI	SGKGT	SVMNTST	8	2	–	2	7	1^a^	2	6	1
3	Wild	LEEAS	NFSND	AICNI	SAKGA	SVMNTNT	3	–	–	3	–	–	–	3	
5	Wild	LEAAS	NFSND	ANRNI	AGKAA	CVIETNT	5	–	–	3	2	–	1	5	
5	Wild	LKAAS	YYSND	ANRNI	AGKAA	SVMNTNT	4	1	1	3	1	–	–	3	–
21	Wild	LEAAS	YFSND	AIRNI	SGKGT	SVMNTST	15	6	1	5	12	3	4	15	3
8	Wild	LEEAS	YFSND	AIRNI	AGKAA	SVMNTNT	6	2	–	2	4	2	1	6	1
3	A675V	LEAAS	NFSND	ANRNI	AGKAA	CVIETNT	2	1		1	2	–	1	–	3
14	Wild	LEAAS	YFSND	ANRNI	SGKGT	CVIETNT	11	3	–	5	7	2	–	10	2

Underlined codons are mutant codons. ETF and LTF stand for early treatment failure and late treatment failure, respectively, whereas S, and RS stand for sensitive, and reduced sensitivity, respectively. Parasite clearance half-life was denoted as PC_1/2_.

^a^Patients did not present adequate plasma dihydroartmisinin concentration, therefore not considered as true day-3 positive case.

### Plasma availability of dihydroartemisinin

We measured artemisinin exposure in patients through detection of plasma DHA. Mean plasma DHA was recorded as 4052 nM, (95% CI 3925.8–4128.1) at 1.5 h and 2137.5 nM, (95% CI 2069.1–2194.8) at 3 h after ASSP exposure. Among 180 patients, 178 patients (98.8%) attained adequate plasma DHA level.

### ACT efficacy

We observed persistence of parasite after 72 h of ASSP exposure in 9 patients (5%), with high median axillary temperature of 38 °C (95% CI 37.8–38.2 °C), corresponding patients also showed prolonged median PC_1/2_ of 5.6 h (95% Cl 5.5–5.7 h). Among them, 8 patients represented adequate plasma DHA level. Of them, 7 patients showed reduced sensitivity to DHA (in vitro) and designated as early ACT failure cases.

We also observed reappearance of infections in 12 patients (6.7%) during 42-days follow-up. After PCR correction through analysing the *msp1, msp2,* and *glurp* genes, we further confirmed the existence of 10 (5.5%) true recrudescence cases among 12 parasite reappearance cases. Crude cure-rate (PCR uncorrected) after ASSP therapy was recorded 89.44% (Kaplan–Meier estimate; 95% CI 83.78–93.35) whereas PCR adjusted cure-rate after day 42 was recorded 90.56% (95% CI 85.07–94.23). We found artemether-lumefantrine (AMLF) rescue therapy was successful without any report of treatment failure (Tables 3, 6).

**Table 3:** Summary of treatment After ASSP therapy.

PCR	Drug	Study population (n)	Day-3 positive parasite	ETF (n)	LTF (n)	ACPR (n)	Recrudescence (n)	Re-infection (n)
PCR uncorrected	ASSP	180	9 (5%)	7^a^ (3.9%)	12 (6.7%)	159 (88.3%)	–	–
PCR corrected	ASSP	180	9 (5%)	7^a^ (3.9%)	10^b^ (5.6%)	161 (89.4%)	10 (5.6%)	2 (1.1%)

ACPR, ETF and LTF stand for adequate clinical parasitological response, early treatment failure and late treatment failure, respectively, whereas ASSP stand for artemisinin-sulfadoxine-pyrimethamine.

^a^One patient did not attain the adequate plasma DHA concentration and in another patient, we failed to adapt the in vitro culture for RSA assay.

^b^Initially reappearance of infection was observed in 12 patients (6.7%), but analyses of *msp1, msp2,* and *glurp* gene confirmed that among those 12 patients, 10 (5.5%) were true recrudescence (LTF) case.

### In vitro drug susceptibility

156 (86.7%) clinical isolates were adapted for their in vitro susceptibility to DHA, sulfadoxine, and pyrimethamine (Table 4). We observed reduced sensitivity to DHA in 11 (7%) isolates. Of those, 7 (77.8%) belonged to VSCP phenotype (mean RSA_(0–3 h)_ = 2.8%; 95% CI 2.4–3.1) and 4 (7.7%) belonged to DCP phenotype (mean RSA_(0–3 h)_ = 1.9%; 95% CI 1.8–2) (Table 4). Lower mean RSA_(0–3 h)_ was recorded in RCP (0.2%; 95% Cl 0.1–0.3) and PCN phenotype (0.2%; 95% CI 0.2–0.3), and were highly sensitive to DHA (p = 0.092).

**Table 4:** In vitro drug susceptibility in different parasite phenotype.

Different parasite phenotype	PC_1/2_ > 5 h	Culture adaptation	RSA (0–3 h) (mean)		IC_50_ nMol/L pyrimethamine (mean)			IC_50_ nMol/L sulfadoxine (mean)		
			Sensitive < 1%	RS ≥ 1%	S < 100 nM	IR 100–2000 nM	R > 2000n M	S < 640 nM	IR 640–3000 nM	R > 3000 nM
Rapid clearing parasite (PCT ≤ 36 h)	0/47	45/47	0.19% (95% CI 0.14–0.25) 45/45	–	72.43 (95% CI 55.8–88) 12/45	1161.25 (95% CI 523.7–1794.2) 17/45	2512.82 (95% CI 2050.2–29.72) 16/45	372.24 (95% CI 236.3–510.1) 21/45	2271.41 (95% CI 1685.4–2860) 14/45	3570.27 (95% CI 3149.2–3987.7) 10/45
Parasite clearance normal (PCT > 36 h ≤ 48 h	0/72	62/72	0.23% (95% CI 0.15–0.32) 62/62	**–**	77.21 (95% CI 64.5–91) 8/62	1330.62 (95% CI 767.3–1891.4) 25/62	4696.70 (95% CI 2675.1–6720) 29/62	426.29 (95% CI 291.4–564.3) 12/62	2349.60 (95% CI;1821.5–2875.8) 28/62	5642.51 (95% CI 4276.2–7012.7) 22/62
Delayed clearing parasite) (PCT > 48- ≤ 72 h)	8/52	41/52	0.33% (95% CI 0.22–0.46 37/41	1.92% (95% CI 1.83–2.02) 4/41	88.9 (95% CI 88–90) 2/41	1741.35 (95% CI 1562.8–1920.5) 12/41	7442.39 (95% CI 5486–9400.2) 27/41	412.54 (95% CI 408.1–417.2) 3/41	2718.50 (95% CI 2635.5–2804.1) 6/41	8156.17 (95% CI 6480.7–9832.3) 32/41
Very slow clearing parasite PCT > 72 h	9/9	8/9	0.94% 1/8	2.76% (95% CI 2.43–3.09) 7/8	**–**	1887.45 1/8	8004.17 (95% CI 6780.3–9235) 7/8	**–**	**–**	8397 (95% CI 7117.2–9680.6) 8/8
3D7			0.17% (95% CI 0.14–0.19	–	68.42 ± 2·1	**–**	–	–	**–**	4320.50 ± 32.65

Individual ex-vivo ring stage survivability of parasite isolates was represented as RSA. Here S, IR, R and RS stand for Sensitive, Intermittent resistant, Resistant, and Reduced susceptibility, respectively. Parasite clearance time was denoted as PCT and Parasite clearance half-life was denoted as PC_1/2_.

We observed prevalence of SP resistant parasite. 79 (50.6%) clinical isolates were identified as pyrimethamine resistance whereas 72 (46.1%) isolates were sulfadoxine resistant (Supplementary Fig. 1A, Supplementary Table 1). Only 22 (14.1%) isolates were sensitive to pyrimethamine and 36 (23.1%) were sensitive to sulfadoxine. Two microscopists showed good conformity during assessment of slides (Pearson correlation r = 0.81, p < 0.005).

### Parasite genetic architecture in relation to parasite clearance outcome

Only 9 isolates (5%) represented *kelch13* mutation (Table 2). Substitution of alanine to valine at codon 675 was observed in 3 (1.7%) isolates (GenBank-N534312-N534314) (Supplementary Fig. 1B), corresponding isolates showed substantially higher RSA_(0–3 h)_ (mean, 2.1%; 95% CI 1.9–2.3) (Table 2). Of those, one patient showed PC_1/2_ of 5.3 h while the remaining represented PC_1/2_ of 4.6 h and 4.9 h, respectively. Of whom, 1 was further developed recrudescence (Table 2). Isolates representing *kelch13-A675V* mutation strongly associated with reduced ex-vivo artemisinin-sensitivity but not showing any relationship in treatment outcome (P = 0.7, Kruskal–Wallis test) Supplementary Fig. 1C). Another 6 (3.3%) patients contained N29L polymorphism, representing lesser PC_1/2_ (2.6 h, 95% Cl 2.4–2.8 h) along with substantially lower RSA_(0–3 h)_ (0.2%; 95% CI 0.1–0.2). Surprisingly, we observed the prevalence (16/17) of prolonged PC_1/2_ (> 5 h) related with wild *pfkelch13* genotype (Table 2).

We found 11 patients (6.1%) with A623E mutation in *pfatpase6* gene (Supplementary Fig. 1B). Of those, two isolates showed PC_1/2_ > 5.5 h. One of the corresponding isolates presented RSA_(0–3 h)_ of 3.3% while others failed to culture adaptation. The isolates containing A623E mutation were sensitive to DHA (0.7%; 95% CI 0.1–1.2) (Supplementary Table 1). Another 5 isolates (2.8%) contained *pfatpase6-*E431K polymorphism (L_263_K_431_A_623_A_630_S_769_), one of those isolates showed PC_1/2_ of 5.1 h (Table 2). Among 5 isolates, three were culture adapted and all were found sensitive to DHA (0.4%; 95% CI 0.4–0.5) (Table 2). Isolates contained polymorphism in *pfkelch13* (N29L, and A675V) and *pfatpase6* gene (E431K, and A623E) represented low to moderate PC_1/2_ (3.9 h; 95% CI 3.5–4.3) but not associated with early ASSP failure (ETF) (P = 0.74, Kruskal–Wallis test).

We observed prevalence of *pfmdr*1-N86Y (65%) and Y184F (50%) mutation (Supplementary Fig. 1D). A total of 48 patients represented Y_86_F_184_S_1034_N_1042_D_1246_ mutation. Of those, 11 (22.9%) showed PC_1/2_ > 5.5 h, of whom 6 (12.5%) showed reduced DHA susceptibility. Another 69 patients contained *pfmdr*1-Y_86_Y_184_S_1034_N_1042_D_1246_ mutation. Of those, only four (5.8%) showed PC_1/2_ > 5.5 h. Isolates containing *pfmdr1*-Y_86_F_184_S_1034_N_1042_D_1246_, and Y_86_Y_184_S_1034_N_1042_D_1246_ mutation presented moderate PC_1/2_ (3.7 h; 95% CI 3.4–4.1), but not associated with ASSP failure (P = 0.56) (Table 2). We also observed prevalence of *pfcrt*-K76T (88.3%) and I356T (68.3%) mutations (Supplementary Fig. 1D). Among 37 isolates with *pfcrt*-S_72_V_73_M_74_N_75_T_76_S_326_T_356_ mutation, 8 (21.6%) showed PC_1/2_ > 5.5 h. Of those 4 (10.8%) represented day-3 positive parasites. 40 patients had *pfcrt*-S_72_V_73_M_74_N_75_T_76_N_326_T_356_ mutation; of those, 4 (10%) had PC_1/2_ > 5.5 h and 3 (7.5%) had day-3 positive parasite. Another 22 contained *pfcrt*-C_72_V_73_I_74_E_75_T_76_ N_326_T_356_ mutation, 4 (18.1%) of those showed PC_1/2_ > 5.5 h, of whom 2 (9.1%) resulted day-3 positive parasite (Supplementary Table 2). We observed combination of *pfmdr1* and *pfcrt* (Y_86_F_184_S_1034_N_1042_D_1246_-S_72_V_73_M_74_N_75_T_76_S_326_T_356_, Y_86_Y_184_S_1034_N_1042_D_1246_-S_72_V_73_M_74_N_75_T_76_S_326_T_356_, and Y_86_F_184_S_1034_N_1042_D_1246_-C_72_V_73_I_74_E_75_T_76_N_326_T_356_) mutations associated with delayed-clearing parasite (PCT > 48– ≤ 72 h) (*P* < 0.02), corresponding isolates showed moderate RSA_(0–3 h)_ (0.78%, 95% CI 0.4–1.3) but were not associated with early ASSP failure (P = 0.68, Kruskal–Wallis test).

### Molecular cause of day-3 positive parasite

We identified 9 day-3 positive cases (Table 5). Of these, 8 represented adequate plasma DHA (mean 2073.2 nmol/L, 95% CI 1960–2180) after 3 h of ASSP exposure, corresponding isolates showed prolonged PC_1/2_ (mean 5.6 h, 95% CI 5.5–5.7) which were significantly higher as compared to PC_1/2_ (3.2 h; 95% CI 2.8–3.6) of cured patients (P < 0.005, Mann–Whitney U-test). Of whom 7 represented significantly higher RSA (mean-2.2%, 95% CI; 1.7–2.8) than the cured patients (mean-0.4%; 95% CI 0.2–0.6). Perhaps these isolates were early ASSP failure cases. The mean axillary temperature [37.9 °C (95% CI 37.8–38.2 °C)] at day three was found very high in those 9 day-3 positive cases. Presence of parasites on day-3 was determined through microscopy as well as PCR-based detection. The mean day-3 parasite load was found 9.1% (95% CI 6.3–11.9%) from that of day-0 parasitemia All day-3 positive isolates had *pfcrt*-I356T polymorphism, while eight of them represented *pfmdr1* N86Y and Y184F double mutations. None of the day-3 positive isolates carried mutation in *pfkelch13* gene (the most recognized and validated artemisinin-resistance associated gene), but 2 had mutation in *pfatpase6* gene. Day-3 positive parasites showing artemisinin-resistance phenotype without *pfkelch13* mutation suggest *kelch13*-independent artemisinin-resistance.

**Table 5:** Distribution of parasite phenotype and genotypes in day-3 positive parasites.

> 72 h (+) case	Plasma artesunate (nmol/L)		Parasite load on day-0 pd/µL	Parasite load on day-3 pd/µL	Fever on day 0 (°C)	Fever on day 3 (°C)	RSA_(0–3 h)_ (in %)	Treatment response (in h)		*Kelch13* genotype	*Atpase6 *haplotype	*Pfmdr1 *haplotype	*Pfcrt *haplotype
	1.5 h	3 h						PCT	PC_1/2_				
Case1	4023	2361	19,368	237	39.6	38.1	2.4	96	6.2	Wild	LEAAS	YYSND	SVMNTNT
Case 2	4196	2147	8432	96	38.5	38	3.0	102	5.6	Wild	LEAAS	YFSND	CVIETNT
Case 3	3833	2051	21,641	356	38.7	38.1	2.1	108	5.6	Wild	LEAAS	YFSND	SVMNTST
Case 4	4077	1952	70,846	611	38.4	37.8	2.6	108	5.8	Wild	LEAAS	YFSND	SVMNTST
Case 5	4086	2052	56,855	123	38.8	37.5	1.7	96	5.3	Wild	LEAAS	YFSND	SVMNTST
Case 6	3981	2114	11,790	202	40.3	38.2	3.3	120	5.9	Wild	LEEAS	YFSND	SVMNTNT
Case 7	3591	1805	44,181	728	39.8	37.9	–	102	5.7	Wild	LEEAS	YFSND	SVMNTNT
Case 8	955	548	9716	455	38	37.5	0.9	108	5.7	Wild	LEAAS	YYSND	SVMNTST
Case 9	4258	2104	36,847	107	39.5	38	2	114	5.5	Wild	LEAAS	YFSND	CVIETNT

PCT represented Parasite clearance time (in h) and parasite clearance half-life was denoted as PC_1/2_ (in h). We have presented parasite density as PD. Individual ex-vivo ring stage survivability of parasite isolates was represented as RSA_(0–3 h)_. Underline codons were mutant codon. In case 8, RSA_(0–3 h)_ was recorded 0.94% with PC_1/2_ of 5.7 h while case 7 was failed to culture adaptation. The day-3 parasite load was ranging from 1.8 to 14.2% from that of day-0 parasitaemia.

### Molecular characterization of parasite recrudescence

**Figure 3:**
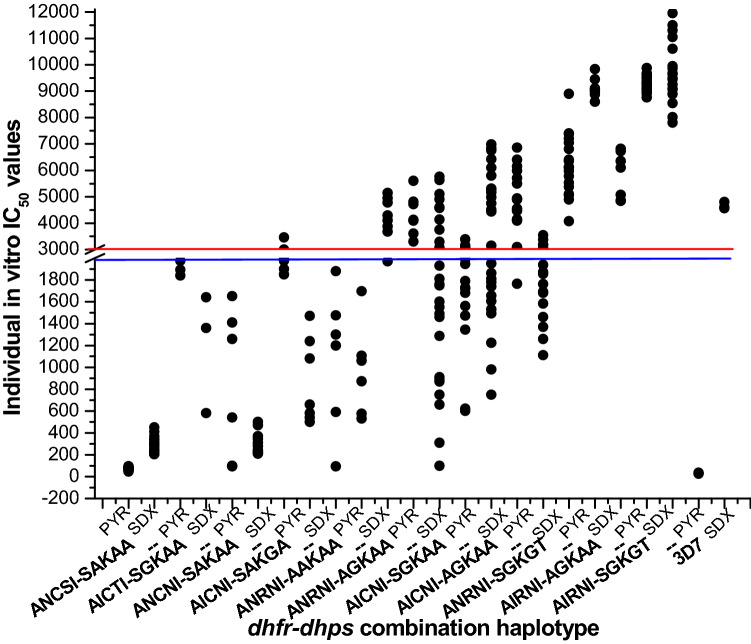
In vitro IC_50_ of pyrimethamine and sulfadoxine in relation to individual dhfr-dhps genotype: Here PYR and SDX respectively stand for “pyrimethamine, and sulfadoxine.” The blue line (corresponding to 2000 nM of PYR) represented the in vitro PYR resistance, while the red line (corresponding to 3000 nM of SDX) represented the in vitro SDX resistance. We observed prevalence of SP resistant parasites. The isolates presenting triple *dhfr* and *dhps* mutation (AIRNI-SGKGT; ANRNI-SGKGT) and double *dhfr* and *dhps* combination mutation (AICNI-AGKAA) represented very high IC_50_ for pyrimethamine and sulfadoxine and proved to be highly resistant to PYR and SDX (*P* < 0.01). Isolates contained ANRNI-AGKAA, AICNI-SGKAA, and ANRNI-AAKAA mutations exhibited moderate to high IC_50_ for pyrimethamine and sulfadoxine but never connected with recrudescence (P = 0.73). Pyrimethamine sensitive and sulfadoxine resistant 3D7 strain was used as a control strain.

After PCR corrections, we identified 10 cases of recrudescence. The mean parasite load on the day of recrudescence was found 8285/µl (95% CI 2726–13,843). The mean body temperature on the day of recrudescence was very high 38.6 °C (95% CI 38.2–39.0 °C). We observed 3 cases of parasite recrudescence within day-7 and another 7 cases between day-8 to day 42, corresponding isolates representing significantly higher IC_50_s for sulfadoxine and pyrimethamine. Parasite recrudescence was the true cause of late ASSP failure. We noticed combination of triple *dhfr* and *dhps* mutation (A_16_I_51_R_59_N_108_I_164_–S_436_G_437_K_540_G_581_T_613_) was highly correlated with parasite recrudescence (*P* < 0.01), while A_16_N_51_R_59_N_108_I_164_–S_436_G_437_K_540_G_581_T_613_ and A_16_I_51_C_59_N_108_I_164_–A_436_G_437_K_540_A_581_A_613_ mutations also contributed crucial role in parasite recrudescence (Table 6) (Supplementary Fig. 1E). Isolates contained A_16_N_51_R_59_N_108_I_164_–A_436_G_437_K_540_A_581_A_613_, A_16_I_51_C_59_N_108_I_164_–S_436_G_437_K_540_A_581_A_613_, and A_16_N_51_R_59_N_108_I_164_–A_436_A_437_K_540_A_581_A_613_ mutations exhibited moderate to high IC_50_ for pyrimethamine and sulfadoxine but never connected with recrudescence (P = 0.73) (Fig. 3). On the day of parasite recurrence, all the LTFs received a standard dose of AMLF and all showed treatment success after 42 days follow-up (Table 6).

**Table 6:** Phenotypic and genotypic characteristics of late treatment failure and evaluation of AMLF rescue therapy.

Case	Drug	Day of recurrence	Parasite load on recurrence	Fever (°C)	PCR correction	*pfk13* allele	*pfdhfr* allele	*pfdhps* allele	Rescue therapy with AMLF		
									ACPR	ETF	LTF
Case 1	ASSP	5	3642/µL	38.6	Recrudescence	Wild	AICNI	AGKAA	√	–	–
Case 2	ASSP	7	5082/µL	38	Recrudescence	Wild	ANRNI	SGKGT	√	–	–
Case 3	ASSP	21	27,419/µL	39.1	Recrudescence	N29L	AICNI	AGKAA	√	–	–
Case 4	ASSP	11	11,026/µL	39.4	Recrudescence	Wild	AICNI	AGKAA	√	–	–
Case 5	ASSP	7	4957/µL	38.4	Recrudescence	A675V	ANRNI	AGKAA	√	–	–
Case 6	ASSP	28	870/µL	37.9	Recrudescence	Wild	AIRNI	SGKGT	√	–	–
Case 7	ASSP	35	7350/µL	38.1	Recrudescence	Wild	ANRNI	SGKGT	√	–	–
Case 8	ASSP	32	512/µL	38.3	Recrudescence	Wild	ANRNI	AGKAA	√	–	–
Case 9	ASSP	28	51,154/µL	40.1	Re-infection^a^	Wild	AIRNI	SGKGT	√	–	–
Case10	ASSP	21	16,822/µL	39.3	Recrudescence	Wild	AIRNI	AGKAA	√	–	–
Case11	ASSP	35	3092/µL	38	Re-infection^a^	Wild	AIRNI	SGKGT	√	–	–
Case12	ASSP	14	5168/µL	38.8	Recrudescence	Wild	AIRNI	SGKGT	√	–	–

Underlined codons are mutant codons. ACPR, ETF and LTF respectively stand for adequate clinical parasitological response, early treatment failure and late treatment failure, whereas ASSP stand for artesunate-sulfadoxine-pyrimethamine and AMLF stands for artemether-lumefantrine. Parasite load was expressed as number of parasite/ micro liter of blood.

^a^After PCR correction, case 9 and case 11 were identified as the case of parasite re-infection.

## Discussion

Emergence and spreading of partial artemisinin resistant parasites in eastern India^27,28^ along with late ACT failures in north-east India^34^ called for a systematic screening of ASSP in central India, as the second highest number of malarial infections was reported from the state of Chhattisgarh. We found the day-3 positive parasite with prolonged parasite clearance half-life (> 5.5 h), along with recrudescence cases. 9.4% of patients showed prolonged PC_1/2_ (> 5.5 h) which was an alarming sign. Prolonged parasite clearance was an indicator of decreased efficacy of fast acting artemisinin within ACT which perhaps led to ACT failure^12,16,19^. We observed the median PC_1/2_ of very slow-clearing parasite (VSCP) (5.6 h) and delayed-clearing parasite (4.2 h) phenotype were significantly higher from the PC_1/2_ of Thailand-Myanmar border (3.7 h) and lower from Western Cambodia (5.9 h)^16^. Identification of these VSCP parasite phenotypes, confirmed the emergence of parasites that became less sensitive to artemisinin in vivo^12^. In spite of this, we also observed isolates with reduced sensitivity to DHA in vitro. The corresponding isolates of VSCP phenotype (5%) represented higher RSA_(0–3 h)_ (2.2%) than the cured patients (0.46%), which proved those VSCP phenotypes were less sensitive to artemisinin in vitro. Previous reports from Thailand suggested that increased viability of ring-stage parasites (RSA_(0–3 h)_ > 1%) was strongly associated with elevated PC_1/2_^16^. Recent reports suggested that apart from reduced efficacy of fast acting artemisinin drugs, failure in late-acting combinations also contributes towards the increment of PC_1/2_^14^. Thus, the reduced artemisinin sensitivity along with elevated PC_1/2_ of the ring stage parasite resulted in reduced sensitivity to ASSP combination therapy. However, most studies in India reported no evidence of less susceptibility to artemisinin in vivo or in vitro^34–36^. Some recent studies reported the spreading of parasites with reduced sensitivity to artemisinin in vivo in eastern India^27,28^. We have identified 9, day-3 positive cases; among them, 7 (3.9%) patients were confirmed as ETF whereas 10 (5.6%) patients were identified as true recrudescence (LTF) cases (Table 2). Given the results of our study, although the numbers of treatment failure cases were not very high, we confirmed the emergence of reduced susceptibility to ASSP combination in this part of India. Nevertheless, findings of reduced efficacy of ASSP in Central India suggested the possibility of emergence of resistant parasites in the near future.

ETF generally occurs due to reduced efficacy of fast-acting artemisinin in combination therapy^18^. The mechanism behind the reduced susceptibility to artemisinin is not fully clear, but genome wide analyses, *ex-vivo* RSA, and transfection studies suggested that artemisinin resistance is predominantly related to *pfkelch13* gene polymorphism^14,18–22^. Interestingly, predominance of wild *pfkelch13* allele was observed in this parasite population of Central India. Despite the direct correlation with *pfkelch13* polymorphism, we had identified 7 (3.9%) ETF cases with prolonged PC_1/2_ as well as increased ex-vivo RSA_(0–3 h)_ survivability, without *pfkelch13* polymorphism. However, the findings of our study were quite uncommon. Our observations from this study suggest several novel aspects of artemisinin-resistance. Firstly, the *kelch13*-indpendent artemisinin-resistance which was previous reported only in Thailand^24,37,38^. Although, we identified 3 isolates with *pfkelch13*-A675V polymorphism which showed positive association with increased ex-vivo RSA_(0–3 h)_, but not with treatment outcome like previous reports from in north-east India and southern Rwanda^39,40^. The results do not represent outliers, as we studied a statistically valid number of patients. Secondly, the absence of *pfkelch13* polymorphism suggests definitive roles for other genetic factors in reduced artemisinin-sensitivity and emergence of artemisinin-resistance. Of note, polymorphism in Ca^2+^ATPase*6* gene (*pfatpase6*) had some role in reducing susceptibility to artemisinin^22^. However, the likelihood of this mechanism has been debated^41^. Likening the observations from Thai-Cambodian border^12^, we have not so far found any definitive correlation between *pfatpase6* mutation and ASSP efficacy In India. Thirdly, *pfcrt, pfmdr,* and *pffd* polymorphisms plausibly represent the genetic background required for the onset of *pfkelch13* polymorphism, as mutations in those genes showed strong association with the beginning of *pfkelch13* mutation during selection of artemisinin-resistance^18,23,27,28^. Indeed, in our study, day-3 positive cases demonstrated acquired mutations in *pfcrt* (K76T, I356T) and *pfmdr1* (N86Y, Y184F) genes similar to the findings reported in eastern and north-India^27,37^. A recent report showed *P. falciparum* strains in South-East Asia having some genetic attenuation to develop novel mutations that caused artemisinin-resistance^42^. Fourthly, the precise genetic architectures in relation to reduced artemisinin-sensitivity in the East, North-East, South-West, and Central India are plausibly different owing to extensive variations in socio-demographic, environmental, seasonal and parasite-vector relationships^26,27,39^.

We detected 10 recrudescence cases in our study. The clinical manifestations of recrudescence were related with less susceptibility of longer acting partner drug (SP), as evidence, parasite isolates showed acquired combination mutation in *pfdhfr*–*pfdhps* gene^8,27,30^. Isolates representing sextuple or quintuple *dhfr*–*dhps* combination mutations (A_16_I_51_R_59_N_108_I_164_–S_436_G_437_K_540_G_581_T_613_, A_16_N_51_R_59_N_108_I_164_–S_436_G_437_K_540_G_581_T_613_ and A_16_I_51_C_59_N_108_I_164_–A_436_G_437_K_540_A_581_A_613_), exhibited very high IC_50_s for pyrimethamine and sulfadoxine, proving true resistance towards SP. Quadruple *dhfr*–*dhps* combination mutations with reduced SP sensitivity was previously reported from Chhattisgarh^29^. Despite the high prevalence of molecular markers associated with SP resistance, treatment failure rate especially LTFs were much less in number. The sensitivity of artesunate over this parasite population was still very high. Artemisinin derivatives within the ASSP were therefore able to kill most of the parasite and reduce the burden of partner drugs. That is how all treatment failure cases recovered after AMLF therapy suggesting that a standard six-dose-AMLF could be a potential second-line treatment against three-dose ASSP failure.

In conclusion, emergence of reduced sensitivity to artemisinin and predominance of SP resistant parasites suggested us for the evaluation of national drug policy as artemether-lumefantrine could be successor of ASSP. Finally, a National Malaria Eradication Program requires urgently for centralized and synchronized implementation of new drug-combinations and tracking of genetic mutations that might lead to higher level of resistance to artemisinin and its partner drug.

## Methods

### Study population

We conducted the study at Bhilai (21.21° N, 81.38° E) and Durg (21.19° N, 81.28° E), districts of Chhattisgarh, India during August 2015–January 2017. Chhattisgarh had contributed the second highest malaria incidence in 2014^9^. The inclusion criteria were age ≥ 3 years, axillary temperature ≥ 37.5 °C, shivering, headache during the past two 2 days or more, and no recent history of anti-malarial medication. All participants were screened for *P. falciparum* infection by microscopic examination of Giemsa-stained thick and thin blood smears. Quantification of parasitaemia was performed by counting the number of parasites per 8000 WBC. We usually count at least 200 fields using 100× oil immersion objectives. Every field generally comprises 4–5 WBCs. Parasites/µl blood was determined as (parasites/WBCs) × 8000 (WBC count/µl of blood). The minimum detectable parasitemia was 40 parasites/µl of blood. *pfmspI* (MAD20 and K1) and *pfmspII* (3D7 and FC27) alleles were screened to detect the clonality of infection. Patients having signs and symptoms of severe malaria, pregnant women, infants and poly-clonal *P. falciparum* infections were excluded^43^. The experimental design and protocols were duly approved by Vidyasagar University, Human ethical committee (VU/HEC 15-0051). We strictly followed the WHO and WWARN (WorldWide Anti-malarial Resistance Network) guideline along with the Helsinki protocol. We obtained duly signed informed consents from each patient and patient’s guardian for minor (child patient) (Fig. 1 for patient selection details).

### ASSP efficacy

Those patients who fulfilled the inclusion criteria received the quality assured standard ASSP dose (supplied by Ministry of Health and Family-Welfare) of 4 mg/kg body weight artesunate once daily for 3 days and a single dose of 25 mg/kg body weight sulfadoxine along with 1.25 mg/kg bodyweight pyrimethamine on first day, under the supervision of medical officer^9^. Trained microscopists checked the thin blood smears at an interval of 6 h, until the patients became parasite free. We estimated the PC_1/2_ by parasite clearance estimator^15^. Patients who developed renal failure and those who vomited the drug were withdrawn from the study and sent for further care. We performed the follow-up evaluations on day 7, 14, 21, 28, 35, and 42 days after initial therapy, and responses were classified accordingly^43^. We performed unscheduled follow-up when symptoms of malaria reappear. Patients, not responding to ASSP, received artemether-lumefantrine rescue therapy (6 tablets, each containing 40 mg AM and 240 mg LF) and were further followed-up for next 42 days.

### Plasma artemisinin testing

We collected 500 µl of intra-venous blood just before, at 1.5 h and 3 h (± 30 min) after initiation of ASSP therapy. We obtained plasma samples immediately and stored at – 20 °C. We evaluated the plasma dihydro-artemisinin (DHA) to validate the sporadic drug exposure by liquid chromatography as stated previously^44^.

### In vitro drug sensitivity testing

We adapted *P. falciparum* clinical isolates in vitro as described previously^28,45^. After culture adaptation parasites were allowed to proliferate for 72 h before doing the anti-malarial drug (sulfadoxine, pyrimethamine, and chloroquine) exposure. We performed sensitivity testing of anti-malarial by hypoxanthine incorporation assay in triplicate, according to our standard laboratory protocol^46,47^. As following the standard guideline, we defined criteria for sulfadoxine, IC_50_ < 640 nM, susceptible; IC_50_ > 640– ≤ 3000 nM, intermediate; and IC_50_ > 3000 nM, resistant. For pyrimethamine, we defined IC_50_ < 100 nM, susceptible; IC_50_ > 100– ≤ 2000 nM, intermediate; and IC_50_ > 2000 nM, resistant. For chloroquine, we defined IC_50_ < 100 nM, susceptible; and IC_50_ > 100 nM, resistant. We used pyrimethamine sensitive and sulfadoxine resistant 3D7 strain as quality-control strain.

### Ring-stage survival testing

We performed ring-stage survival **(**RSA) assay in triplicate after culture adaption of clinical isolates as described earlier^17^. We treated 0–3 h post-invasive, highly synchronized early ring-stage parasites with 700nMoles of dihydro-artemisinin for 6 h, followed by washing with RPMI-1640 for three times and further cultivated for another 66 h. We measured parasite survival rates by microscopic examination of 10,000 RBCs per treatment replicate in Giemsa-stained thin blood smears.

### DNA extraction and sequencing

We extracted parasite DNA from frozen blood aliquots (200 µl) using Mini blood-kit (Qiagen). We performed nested PCR to amplify *pfkelch13* gene using *Kelch13*-F and *Kelch13*-R primers according to our standard laboratory protocol^28^. We also performed nested PCRs to amplify *pfATPase6, pfdhfr, pfdhps, pfmdr1,* and *pfcrt* gene as described previously^46–48^*.* For *pfATPase6* gene amplifications, we used primer-pairs (5′TTGGTAATAAAACTCCCGC3′ and 5′TATTCCTCTTAG-CACCACTCC3; for 250–500 codon; 5′AAGAAGGATAAATCACCAAG3′ and 5′AAATACACGTATA-CCAGCC3′; for 520–800 codon). We sequenced the amplicons directly using the BigDye Terminator v3.1 Sequencing Kit (Applied Biosystems), and were run on 3730xl genetic analyzer^48^. We used online translation tool (http://www.expasy.org) to translate the sequences. We queried single nucleotide polymorphism of individual sequences by using a nucleotide database with BLAST.

### Statistical analysis

We expressed our data as a univariate median; mean ± SEM. Fisher’s exact test along with regression analyses were performed to correlate the treatment efficacies with molecular genotyping. We used the Clopper-Pearson method to calculate the 95% confidence intervals. Data were compared between two groups by Mann–Whitney U-test while Kruskal–Wallis-test was used to compare among more than two groups. We considered p < 0.05 statistically significant. All statistical analyses were performed through Graph Pad in-Stat 3.0 and Origin 6.1.

## Supplementary Information

Supplementary Information.
