# Supplementary material for: Artemisinin combination therapy fails even in the absence of Plasmodium falciparum kelch13 gene polymorphism in Central India
Source: Sci Rep. 2021 May 11;11:9946. doi: 10.1038/s41598-021-89295-0 (PMC8113598; doi:10.1038/s41598-021-89295-0)
Supplement: Supplementary file 1 — Supplementary Information. [file 41598_2021_89295_MOESM1_ESM.docx]

**Title:** **Artemisinin combination therapy fails even in the absence of *Plasmodium falciparum kelch13* gene polymorphism in Central India.**

**Author: Sabyasachi Das,^1,2,3*^ Amrita Kar ,^4^ Subhankar Manna,^3^ Samaresh Mandal,^3^ Sayantani Mandal,^5^ Subhasis Das,^5^ Bhaskar Saha,^6^ Amiya Kumar Hati ^7^.**

^1^ Department of Physiology, Faculty of Medicine, Melaka Manipal Medical College University, Melaka, Malaysia

^2^ Department of Physiology, Faculty of Medicine, Lincoln University College, Malaysia

^3^Department of Human Physiology, Vidyasagar University, West Bengal, India.

^4^ Department of Biotechnology, Sastra University, Thanjavur, Tamil Nadu, India.

^5^ Medicare, Bhilai, Chhattisgarh, India.

^6^Infection and Immunity lab, National Centre for Cell Science, Ganeshkhind, Pune, India.

^7^ Department of Medical Entomology and Parasitology, Calcutta School of Tropical Medicine, West Bengal, India.

**Running title**: ACT treatment failure without *kelch13* mutation

***Address of correspondence:**

###### Dr. Sabyasachi Das,

###### Assistant Professor, Department of Physiology,

###### Faculty of Medicine, Manipal University College Malaysia.

**Jalan Batu Hampar, Bukit Baru, 75150Melaka**

**Mob: +60 1161 908 761 E-mail: [sdas.vu@gmail.com](mailto:sdas.vu@gmail.com)**

[**Sabyasachi.das@edu.my**](mailto:Sabyasachi.das@edu.my)

**Supplementary Table 1:**  Distribution of candidate gene haplotype in relation to in vitro susceptibility to chloroquine, artesunate, pyrimethamine and sulfadoxine

| No of Isolates | Culture Adapted | Kelch 13 genotype | ATPase6 (263+4 31+623+ 630+769 | Pfmdr1 (86+184+ 1034+ 10 42+1246 | Pfdhfr (16+51+ 59 +108 + 164) | Pfdhps (436+437+540+581+ 613) | Pfcrt  (72-76 +326+356) | AS (RSA) sensitivity | | PYR sensitivity | | | SDX sensitivity | | | CQ sensitivity | |
| --- | --- | --- | --- | --- | --- | --- | --- | --- | --- | --- | --- | --- | --- | --- | --- | --- | --- |
|  |  |  |  |  |  |  |  | S | RS | S | IR | R | S | IR | R | S | R |
| 21 | 18 | Wild | LEAAS | NYSND | ANCSI | SAKAA | CVMNKNI | 18 | - | 18 | - | - | 18 | - | - | 18 | - |
| 11 | 10 | Wild | LEAAS | YYSND | ANCNI | SAKAA | CVMNTNI | 10 | - | 2 | 4 | 4 | 10 | - | - | 3 | 7 |
| 6 | 4 | Wild | LEAAS | NFSND | AICNI | SAKGA | SVMNTSI | 4 | - | - | 2 | 2 | 2 | 2 | - | - | 4 |
| 16 | 14 | Wild | LEAAS | NFSND | AICNI | SGKAA | SVMNTSI | 14 | - | - | 7 | 7 | 1 | 11 | 2 | - | 14 |
| 7 | 6 | Wild | LEAAS | YYSND | ANRNI | AAKAA | SVMNTNT | 6 | - | 1 | 5 | - | 2 | 4 | - | - | 6 |
| 24 | 21 | Wild | LEAAS | YYSND | AICNI | AGKAA | SVMNTNT | 20 | 1 | 1 | 8 | 12 | - | 10 | 11 | - | 21 |
| 3 | 3 | Wild | LEAAS | NFSND | AICTI | SGKAA | SVMNTSI | 3 | - | - | 3 | - | 1 | 2 | - | - | 3 |
| 17 | 16 | Wild | LEAAS | YYSND | AICNI | SGKAA | CVIETNT | 16 | - | - | 8 | 8 | 1 | 11 | 4 | - | 16 |
| 6 | 6 | N29L | LEAAS | NFSND | AICNI | AGKAA | SVMNTST | 6 | - | - | 4 | 2 | - | 3 | 3 | - | 6 |
| 10 | 7 | Wild | LEAAS | YYSND | ANRNI | SGKGT | SVMNTST | 6 | 1 | - | 4 | 3 | - |  | 7 | - | 7 |
| 3 | 3 | Wild | LEEAS | NFSND | AICNI | SAKGA | SVMNTNT | 3 | - | - | 2 | 1 | 1 | 2 | - | - | 3 |
| 5 | 5 | Wild | LEAAS | NFSND | ANRNI | AGKAA | CVIETNT | 5 | - | - |  | 5 | **-** | 2 | 3 | - | 5 |
| 5 | 3 | Wild | LKAAS | YYSND | ANRNI | AGKAA | SVMNTNT | 3 | - | - | 1 | 2 | **-** | 1 | 2 | - | 3 |
| 21 | 18 | Wild | LEAAS | YFSND | AIRNI | SGKGT | SVMNTST | 15 | 3 | - |  | 18 | **-** | - | 18 | - | 18 |
| 8 | 7 | Wild | LEEAS | YFSND | AIRNI | AGKAA | SVMNTNT | 6 | 1 | - |  | 7 | - | - | 7 | - | 7 |
| 3 | 3 | A675V | LEAAS | NFSND | ANRNI | AGKAA | CVIETNT |  | 3 | - |  | 3 | - | - | 3 | - | 3 |
| 14  Pyrimethamine was represented as PYR while sulfadoxine was denoted as SDX. Chloroquine was represented as CQ. Underline codons were mutant codon. Individual ex-vivo ring stage survivability of parasite isolates was represented as RSA. | 12 | Wild | LEAAS | YFSND | ANRNI | SGKGT | CVIETNT | 10 | 2 | - | 7 | 5 | - | - | 12 | - | 12 |

**Supplementary Table 2: Individual parasite haplotype and their association with in vivo and in vitro parasite phenotype.**

| **Gene** | **Allele** | **Number** | **PCHL** | **DCP (48h-72h)** | **ETF; 72h+ case** | **RS of DHA through RSA** |
| --- | --- | --- | --- | --- | --- | --- |
| *Pfkelch 13* | Wild | 171 | 16 (9.35%) | 49 (28.65%) | 9 (5.26) | 8 (4.67) |
|  | N29L | 6 | 0 (0.00%) | 1 (16.67%) | 0 (0.00%) | 0 (0.00%) |
|  | A675V | 3 | 1 (33.33%) | 2 (66.67%) | 0 (0.00%) | 3 (100%) |
| *Pfatpase6* | Wild LEAAS | 164 | 14(8.53%) | 47 (28.66%) | 7 (4.26%) | 10 (6.09%) |
|  | LEEAS | 11 | 2 (18.18%) | 4 (36.36%) | 2(18.18%) | 1(9.09%) |
|  | LKAAS | 5 | 1 (20%) | 1 (20%) | 0 (0.00%) | 0 (0.00%) |
| *Pfmdr1* | Wild NYSND | 21 | 0 (0.00%) | 3 (14.28) | 0 (0.00%) | 0 (0.00%) |
|  | YFSND | 48 | 12 (25.00%) | 24 (50.00%) | 7(14.58%) | 6 (12.5%) |
|  | YYSND | 69 | 3 (4.34%) | 15 (21.74%) | 2 (2.89%) | 2 (2.89%) |
|  | NFSND | 42 | 2 (4.76%) | 10 (23.81%) | 0 (0.00%) | 3 (7.14%) |
| *Pfcrt* | CVMNKNI | 21 | 0 (0.00%) | 3 (14.28%) | 0 (0.00%) | 0 (0.00%) |
|  | CVMNTNI | 11 | 0 (0.00%) | 2 (18.18%) | 0 (0.00%) | 0 (0.00%) |
|  | SVMNTSI | 25 | 1 (4%) | 5 (20%) | 0 (0.00%) | 0 (0.00%) |
|  | SVMNTNT | 47 | 4 (8.51%) | 9 (19.14%) | 3 (6.38%) | 2 (4.25%) |
|  | CVIETNT | 39 | 4 (10.25%) | 13 (33.33%) | 2 (5.13%) | 5 (12.82%) |
|  | SVMNTST | 37 | 8 (21.62%) | 20 (54.04%) | 4(10.81%) | 4 (10.81%) |

**Sup Fig 1D.**

**Supplementary Figure 1**

**Sup Fig 1E.**

**Sup Fig1B**

**SupFig 1A**

**Sup Fig 1C**

**Figure Legends:**

**Supplementary Figure 1 A: In vitro drug sensitivity to artemisinin (RSA assay), pyrimethamine and sulfadoxine**. Here S, IR and R respectively stand for “sensitive, intermediate sensitive and resistant” parasite, whereas RS denotes reduced susceptibility. Regarding artemisinin, survivability of ring-stage parasite >1% represented as reduced susceptibility. Predominance of ART sensitive parasite was found. In case of pyrimethamine, predominance of resistant parasite (50.65%) was observed. On the contrary, only 14.10 % isolates had represented pyrimethamine sensitive parasite and 23.07% isolates were sensitive to sulfadoxine. Prevalence of sulfadoxine resistant parasites (46.75%) were found followed by intermediate resistant parasite (30.77%).

**Supplementary Figure 1 B: Genotype variations of *pfkelch13* and *pfatpase6* gene:** In *pfkelch13* gene predominance of wild type allele was observed. Only 3 isolates represented A675V mutation and another 6 isolated represent N29L mutations, located outside the coding sequence of kelch domain. In *pfatpase6* gene Wild LEAAS allele was prevalent (91.11%) followed by LEEAS (A623E mutation, 6.11%) and LKAAS (E431K mutation, 2.78%) allele. Mutations were absent at L263E, A630S, and S769N codon.

**Supplementary Figure 1C: Proportion of parasite sensitivity in relation to different *kelch13* allele:** The solid line (corresponding to 1% parasite survival after RSA) represented the reduced artemisinin-sensitivity, while the arrows (corresponding to PC_1/2_>5.5h) were showing less efficacy of ASSP in vivo. A significant proportion of wild *kelch13* parasite represented both reduced sensitivity to artemisinin ex vivo as well as representing prolonged PC_1/2_.

**Supplementary Figure 1 D: Haplotype frequency of *pfmdr1* and *pfcrt* gene**. In *pfmdr1* gene, single mutant YYSND allele (38.33%) was most common allele followed by YFSND (26.67%) and NFSND (23.33%) allele. In *pfcrt* gene, triple mutant SVMNTNT allele (26.11%) was most common allele followed by CVIETNT (21.67%) and SVMNTST (20.56%) allele.

**Supplementary Figure 1E: Haplotype frequency of *pfdhfr* and *pfdhps* gene**. In *dhfr* gene, S108N mutation was most common mutation (156, 86.67%) followed by N51I (104, 57.78%) and 59R (73, 40.55%). AICNI (40.0%), ANRNI (24.44%) and AIRNI (16.11%) were frequently found *dhfr* haplotype. On the contrary, predominance of *dhps* A437G (73.33%) mutation was observed after S436A (32.22%) and A580G (30%) mutation. AGKAA.
